# A Perspective on Lanthanide-Containing Nanocomposite Hydrogels: Current Research and Future Directions

**DOI:** 10.1021/acsmaterialsau.5c00125

**Published:** 2025-10-01

**Authors:** Yu-Ning An, Yi-Cheun Yeh

**Affiliations:** Institute of Polymer Science and Engineering, 33561 National Taiwan University, Taipei 10617, Taiwan

**Keywords:** lanthanide, luminescence, nanomaterials, hydrogels, nanocomposites

## Abstract

Lanthanide-containing nanocomposite hydrogels represent a versatile class of functional materials with significant potential for applications in chemical sensing, biomedicine, and information security. Diverse chemical strategies and design methodologies have been employed to tailor their structure–property–function relationships. In this perspective, we provide an overview of recent advancements in lanthanide-containing nanocomposite hydrogels, systematically categorized into five material-centric approaches, including upconversion nanoparticles, metal–organic frameworks, nanoclays, hydroxyapatite, and carbon-based nanomaterials. We summarize key developments in their composition, interfacial chemistry, and applications while also evaluating current challenges and outlining future research directions to guide the continued evolution of these hydrogel systems.

## Graphical abstract

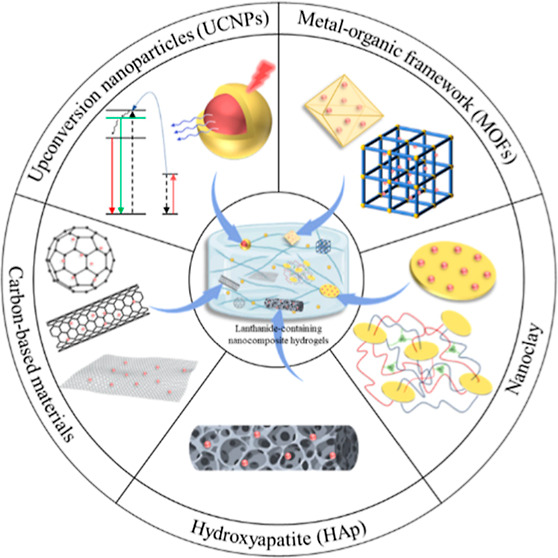

## 1 Introduction

Nanocomposite hydrogels, with the incorporation of nanomaterials into hydrogel networks, present improved mechanical strength, tunable responsiveness, and advanced functionalities as versatile platforms for applications in drug delivery,^1^ sensing,^2^ and tissue engineering.^3^ Lanthanide (Ln)-containing nanocomposite hydrogels have emerged as a promising class of multifunctional materials by combining the unique optical and magnetic properties of lanthanide elements with the structural flexibility of hydrogels and the functional advantages of nanomaterials. In general, Ln-containing nanocomposite hydrogels are composed of three-dimensional, hydrophilic polymeric networks embedded with Ln-doped nanomaterials such as upconversion nanoparticles (UCNPs),^4–7^ metal–organic frameworks (MOFs),^8,9^ nanoclays,^10^ hydroxyapatite (HAp),^11^ and carbon-based nanomaterials.^12^ The 4f electron configuration of lanthanide ions imparts these systems with sharp emission peaks, long luminescence lifetimes, large Stokes shifts, as well as magnetic and catalytic properties.^13^

Compared to conventional Ln-containing hydrogels, where lanthanide ions or complexes are directly introduced into the polymer matrix,^14^ nanocomposite hydrogels offer several advantages by utilizing the structural and functional characteristics of nanomaterials. Traditional systems often suffer from luminescence quenching due to the high water content of hydrogels, which facilitates nonradiative deactivation through vibrational coupling with water molecules.^15^ In contrast, embedding lanthanide ions within nanomaterials reduces such quenching by protecting the ions from an aqueous environment to enhance luminescence intensity and stability. In addition, nanomaterials can act as physical fillers or chemical cross-linkers, leading to improved mechanical properties (e.g., tensile strength and elasticity) and stimuli-responsiveness (e.g., pH, temperature, and light). For example, UCNPs allow near-infrared (NIR)-induced luminescence, while MOFs provide porous structures that support molecule encapsulation, offering capabilities not achievable by lanthanide ions alone.

Ln-containing nanocomposite hydrogels have shown great potential across various domains. For biomedical applications, lanthanide-containing nanocomposite hydrogels generally demonstrate low toxicity and good biocompatibility in both cellular and organismal systems. For instance, the nanocomposite hydrogels with UCNPs coated with diacrylated Pluronic F127 (DA-PF127) maintained high cell viability (98.5%) in NIH/3T3 fibroblast cultures, effectively minimizing nanoparticle leaching and further reducing potential *in vivo* toxicity risks.^5^ By contrast, lanthanides are known to exhibit toxicity in living organisms, which primarily arises from Ln^3+^ ions mimicking calcium (Ca^2+^) and magnesium (Mg^2+^) due to similar ionic radii, as well as lipid peroxidation, phosphate depletion from insoluble lanthanide–phosphate complex formation, and disrupting processes (e.g., neurotransmission, signaling, and enzymatic activity).^16–19^

Therefore, Ln-containing nanocomposite hydrogels have been developed as luminescent probes for high-resolution imaging,^20^ contrast agents for magnetic resonance imaging (MRI),^21^ platforms for controlled drug release,^7^ and tissue repair.^22^ For instance, Leu Alexa et al. reported cerium (Ce^3+^)-doped Hap-containing hydrogels present excellent printability and bone-regeneration capabilities.^23^ For example, Cheng et al. demonstrated hydrogels embedded with sodium yttrium fluoride (NaYF_4_): ytterbium­(Yb^3+^)/erbium (Er^3+^) UCNPs and carbon nanotubes for dual-purpose drug delivery and luminescence labeling,^24^ and Li et al. reported laponite-based hydrogels with lanthanide ions showing pH-responsive reversible luminescence.^14^ For environmental applications, these hydrogels have been used as sensors for detecting heavy metals and organic pollutants.^25–27^ For example, Zeng et al. developed injectable and self-healable luminescent europium­(Eu^3+^)-containing gelatin/polydextran/laponite nanocomposite hydrogels for distinguishing formaldehyde in volatile organic compounds (VOCs).^25^

Early research primarily explored the optical and magnetic behavior of lanthanide ions within hydrogel systems, and several great reviews have summarized the reports of the Ln-containing hydrogels.^28–31^ Nonetheless, current reviews lack systematic classification based on the type of nanomaterials, limiting our understanding of the structure–property relationships that drive functional performance. This perspective aims to address these gaps by categorizing recent developments in Ln-containing nanocomposite hydrogels into five material-focused directions, including UCNPs, MOFs, nanoclay, HAp, and carbon-based nanomaterials (Figure 1). A summary of recent Ln-containing nanocomposite hydrogels, highlighting their compositions, interfacial chemistry, and applications, is provided in Table 1, with selected representative examples further discussed in the following sections.

**Figure 1:**
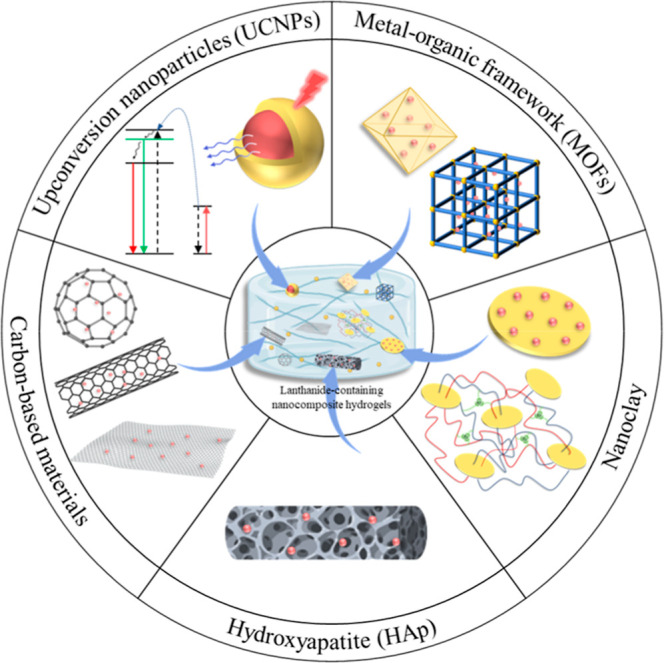
Classification of lanthanide-containing nanomaterials used for the construction of nanocomposite hydrogels.

**Table 1:** Summary of Ln-Containing Nanocomposite Hydrogels Categorized by Nanomaterial Types, Highlighting Their Compositions, Interfacial Chemistry, and Applications

nanomaterial types	lanthanides	Ln-incorporated nanomaterials	polymers	interfacial chemistry	applications	reference
UCNP	Yb^3+^, Tm^3+^, Nd^3+^	NaYF_4_:Yb^3+^,Tm^3+^@NaYF_4_:Nd^3+^,Yb^3+^ (UC-YT@NY) UCNP	gelatin methacrylate (GelMA)	hydrogen bonding (UCNP/GelMA)	deep tissue wound healing	^4^
	Yb^3+^, Er^3+^	NaYF4:Yb^3+^, Er^3+^ UCNP	4-arm polyethylene glycol acrylate (4-arm PEG-AC); diacrylated Pluronic F127 (DA-PF127)	covalent bonding (4-arm PEG-AC/DA-PF127); hydrophobic interaction (UCNP/DA-PF127)		^5^
	Yb^3+^, Er^3+^	NaYF_4_:Yb,Er@NaYF_4_ UCNP	polyacrylamide	UCNPs were embedded in hydrogels via in situ polymerization and further stabilized by hydrogen bonds and van der Waals forces	formaldehyde detection	^6^
	Yb^3+^, Tm^3+^	LiYF_4_:Yb^3+^/Tm^3+^ UCNP	chitosan; polyethylene glycol-bisazide (PEGBA)	UCNPs with a thin shell of chitosan, and the shell was covalently cross-linked with a photocleavable cross-linker (PhL); the azide groups from PEGBA reacted with the acetylene groups of PhL via a click reaction	drug delivery; deep tissue imaging	^7^
	Yb^3+^, Er^3+^	NaYF_4_:Yb^3+^/Er^3+^ UCNP; multiwalled carbon nanotube	poly(*N*-isopropylacrylamide-*co*-acrylamide)	UCNPs were physically embedded in the polymeric network	NIR-triggered drug release; upconversion luminescence tagging for bioimaging	^24^
	Er^3+^,Yb^3+^	NaYF_4_:Er^3+^,Yb^3+^ UCNP	poly(ethylene glycol) diacrylate; *N*-vinylpyrrolidone	the polymers were covalently cross-linked; UCNPs were physically embedded in the polymeric network through hydrogen bonding	controlled drug delivery	^32^
MOF	Eu^3+^	Eu@MIL-116(Ga) MOF	alginate	Ga^3+^ chemically cross-linked alginate	mitoxantrone detection	^9^
	Eu^3+^	methyl red@lanthanide MOF (MR@EuMOF)	carboxymethyl cellulose	MOFs were physically embedded in the polymeric network	histamine vapor detection	^33^
	Eu^3+^, Tb^3+^	Eu@MOF-808-TDA, Tb@MOF-808-TDA (Ln-functionalized MOF-808 via thiodiglycolic acid)	poly(vinyl alcohol)	MOFs were physically embedded in the polymeric network	Al^3+^ and UO_2_^2+^ detection	^34^
	Eu^3+^	Eu_2_(BPDC)(BDC)_2_(H_2_O)_2_ MOF	alginate	MOFs were physically embedded in the alginate hydrogel; Fe^3+^ chemically cross-linked alginate	β-lactamase detection	^8^
	Eu^3+^, Tb^3+^	Eu^3+^/Tb^3+^@Uio-66-(COOH)_2_/NDC MOF	alginate	MOFs were physically embedded in the polymeric network	nitrophenol isomer detection	^35^
nanoclay	Tb^3+^	Tb^3+^-doped laponite (Tb^3+^@Laponite)	polyethylenimine-modified gelatin (PG); polydextran aldehyde (PDA)	imine bonds (PG/PDA); metal–ligand coordinated bonds (Tb^3+^@Lap/PG); hydrogen bonds and electrostatic interactions (PG/PDA/Tb^3+^@Lap)	electrospinning; 3D printing; Cu^2+^ detection	^10^
	Eu^3+^ or Tb^3+^	aminoclay (AC); Ln–ligand (Ln–L) complexes	polyacrylamide (PAAm)	hydrogen bonds (aminoclay/PAAm)		^36^
	Tb/2,6-pyridine dicarboxylic acid complex (Tb·L_3_); Eu/2,6-pyridine dicarboxylic acid complex (Eu·L_3_)	laponite	poly(*N*-isopropylacrylamide); poly(*N*,*N*-dimethylacrylamide)	laponite was physically embedded in the polymeric network	thermal-responsive hydrogel actuator	^37^
	Eu^3+^, Tb^3+^	laponite	poly(*N*-isopropylacrylamide)	laponite was physically embedded in the polymeric network		^38^
	Eu^3+^	Eu-DPA@clay	polyacrylamide	laponite was physically embedded in the polymeric network		^14^
	Eu^3+^	Eu(DPA)_3_@Laponite-Tris (Eu^3+^-coordinated 2,6-pyridinedicarboxylic acid (DPA) on tris-functionalized laponite)	polyvinyl alcohol	laponite was physically embedded in the polymeric network	glutathione detection	^39^
	Eu^3+^	Eu^3+^@Lap (Eu^3+^ ion-exchanged Laponite); Eu^3+^(TTA)@Laponite (2-thenoyltrifluoroacetone (TTA)-modified Eu^3+^@Laponite)	polyethylenimine-modified gelatin (PG); polydextran aldehyde (PDA)	imine bonds (PG/PDA); metal–ligand coordinated bonds (PG/Eu^3+^@Lap; PG/Eu^3+^(TTA)@Lap); hydrogen bonds and electrostatic interactions (PG/PDA/Eu^3+^@Lap; PG/PDA/Eu^3+^(TTA)@Lap)	formaldehyde vapor detection	^25^
HAp	Eu^3+^, Tb^3+^, Dy^3+^	Ln-HAp@L (Ln = Eu, Tb, or Dy; L = nitrilotriacetic acid), di(2-picolyl)amine, or bisphosphonate)	polydextran aldehyde (PDA)	imine bonds (PDA/Ln-HAp@L)	differentiation of volatile organic compounds	^11^
	Ce^3+^, Ce^4+^	Ce-HAp	gelatin methacryloyl (GelMA)	Ce-HAp dispersed in GelMA before photopolymerization	3D printing	^23^
carbon-based nanomaterials	Eu^3+^, Tb^3+^	ethylenediaminetetraacetic acid (EDTA)-functionalized carbon quantum dots	alginate	lanthanide ions interacted with alginate through ionic cross-linking	Ca^2+^ detection	^12^
	Gd^3+^	carbon nanotubes; SiO_2_@Fe_3_O_4_	chitosan	Gd^3+^-imprinted chitosan (IIP-CS) coated on CNTs via surface deposition-cross-linking, forming IIP-CS/CNT; SiO_2_@Fe_3_O_4_ trapped in IIP-CS/CNT network via physical blending	adsorption of Gd^3+^ from solutions	^26^

## 2 Ln-Containing Nanocomposite Hydrogels

### 2.1 Ln-UCNPs

Ln-incorporated UCNPs are a class of luminescent nanomaterials capable of converting low-energy NIR light into higher-energy visible or ultraviolet (UV) emissions.^40^ Composed of a host lattice (e.g., sodium yttrium fluoride (NaYF_4_)^4–6^ or lithium yttrium fluoride^7^) doped with lanthanide ions, UCNPs offer sharp emission bands, long luminescence lifetimes, and excellent photostability. When incorporated into hydrogels, UCNPs enable NIR-responsive functionalities to penetrate biological tissues with minimal thermal damage for deep-tissue photopolymerization,^41^ drug release,^42^ and noninvasive tracking.^43^

UCNPs can be used as photosensitizers to convert NIR light to UV or visible light to initiate the photo-cross-linking between polymers for hydrogel formation under mild conditions. For example, An et al. synthesized core–shell NaYF_4_:Yb^3+^,Tm^3+^@NaYF_4_:Nd^3+^,Yb^3+^ (UC-YT@NY) UCNPs via coprecipitation, coated with poly­(acrylic acid) (PAA) to enhance hydrophilicity, and integrated them with gelatin methacrylate (GelMA) to form porous UC-YT@NY + GelMA nanocomposite hydrogels (Figure 2a).^4^ Specifically, the hydrogel was formed by mixing UC-YT@NY with GelMA and photoinitiator (i.e., lithium phenyl-2,4,6-trimethylbenzoylphosphonate), followed by 808 or 980 nm NIR irradiation, which triggered the UCNP emission of UV light to initiate free-radical polymerization of methacryloyl groups on GelMA. This process enabled deep-tissue curing with minimal thermal effects (Δ*T* < 3 °C), facilitating the transition from a liquid precursor to a solid hydrogel. While UCNPs alone exhibited slight toxicity to cells, the incorporation of GelMA into the hydrogel improved cell viability and maintained normal cell morphology, suggesting that the hydrogel provided a favorable microenvironment for cell growth. Furthermore, *in vivo* experiments demonstrated that these photopolymerized hydrogels could adapt to mouse skin defects and accelerate wound healing, indicating their biocompatibility and efficacy at deeper tissue levels. Overall, the hydrogel promoted wound healing through its biocompatible, porous structure with reactive amino, carboxyl, and hydroxyl groups, which fostered cell proliferation and migration. The 808 nm irradiation further enhanced healing by promoting cell migration, extracellular matrix deposition, and angiogenesis via deeper tissue penetration and mild thermal stimulation.

**Figure 2:**
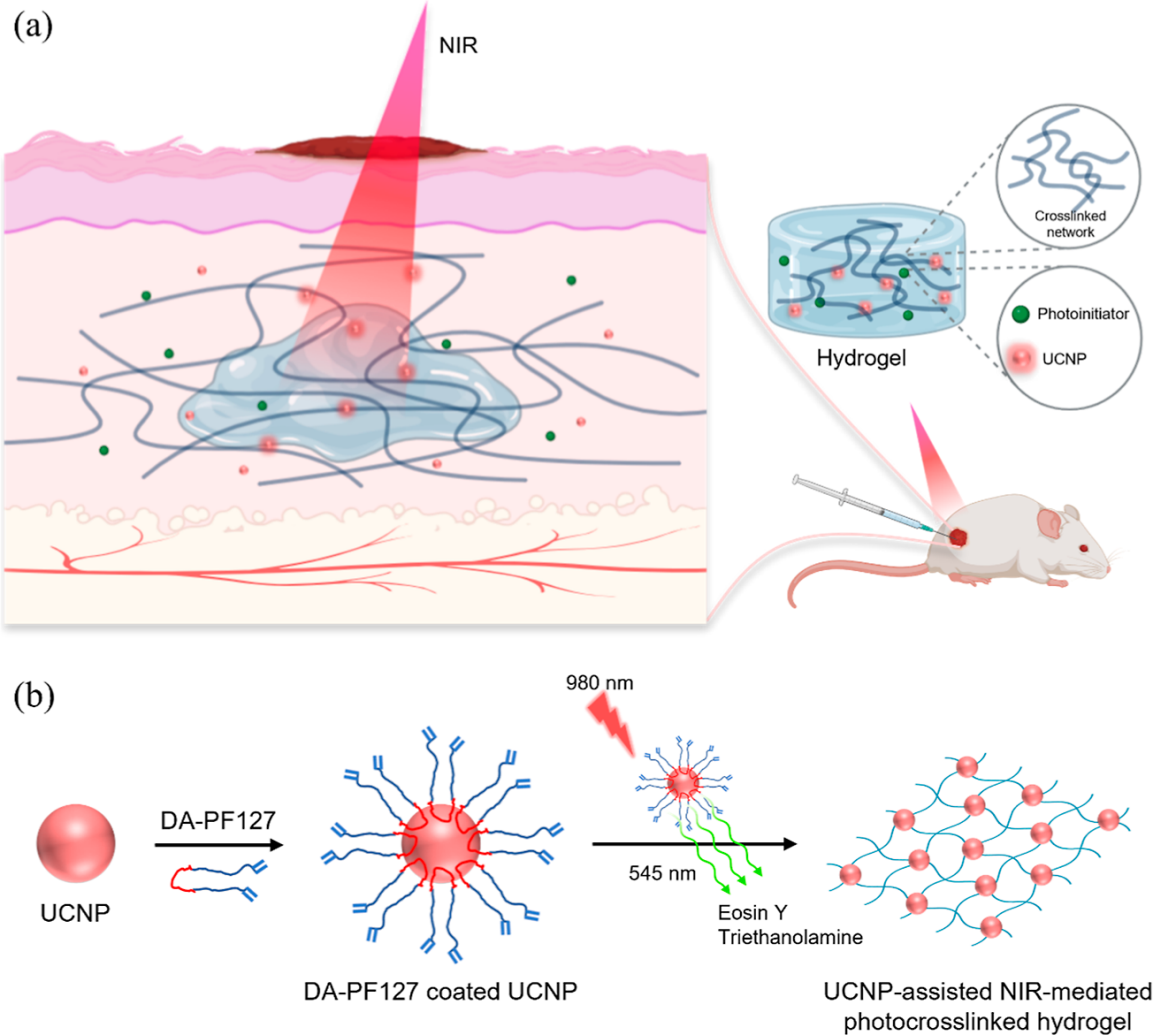
NIR-triggered UCNP-mediated photopolymerization for nanocomposite hydrogel formation. (a) Schematic illustration of *in vivo* wound healing using UC-YT@NY + GelMa hydrogel hybrids, where the NIR irradiation triggered UCNP emission of UV light to initiate polymerization of GelMA to allow deep-tissue curing. (b) Schematic illustration of NIR-mediated photopolymerization employing DA-PF127-coated NaYF_4_:Yb^3+^,Er^3+^ UCNPs. Under 980 nm irradiation, the UCNPs emitted green light that activated the photoinitiator eosin Y (EY) in the presence of the co-initiator triethanolamine to generate free radicals.

In another example, Gwon et al. synthesized NaYF_4_:Yb^3+^,Er^3+^ UCNPs and coated them with DA-PF127 to form a nanocomposite hydrogel via NIR-mediated photopolymerization (Figure 2b).^5^ Under 980 nm NIR irradiation (3 W/cm^2^, 10 min), the UCNPs emitted green light (500–570 nm), which overlapped with the absorption spectrum of EY, activating EY with triethanolamine as a co-initiator to generate free radicals. These radicals initiated the polymerization of acrylate groups from 4-arm PEG-acrylate, *N*-vinylpyrrolidone (NVP), and DA-PF127, with DA-PF127 on UCNPs serving as nucleation sites for cross-linking, enabling the transition from a liquid precursor to a solid hydrogel. The surface modification of UCNPs with DA-PF127 enhanced both photopolymerization efficiency and colloidal stability. The acrylate groups on DA-PF127-coated UCNPs actively participated in free-radical polymerization, functioning as initiation and cross-linking sites that promoted more efficient network formation. As a result, the modified UCNPs yielded stiffer hydrogels with reduced swelling ratios, improving their structural integrity and suitability for biomedical applications. Most importantly, coating the surface of UCNPs with DA-PF127 chemically anchored the nanoparticles within the network to prevent leaching, reducing the potential toxicity associated with UCNPs. Therefore, the in situ formation and biocompatibility of hydrogels make them promising for biomedical applications such as minimally invasive tissue engineering.

UCNPs can act as sensing elements to enhance the detection capabilities of nanocomposite hydrogels. Zhu et al. developed a dual-mode hydrogel nanosensor for formaldehyde detection by embedding NaYF_4_:Yb,Er@NaYF_4_ UCNPs with chromophores within a hydrogel matrix.^6^ The hydrogel was formed using polyacrylamide, a synthetic polymer cross-linked through free-radical polymerization initiated by ammonium persulfate and *N*,*N*,*N*′,*N*′-tetramethylethylenediamine (TEMED), creating a three-dimensional porous network that encapsulated the UCNPs. For detection, the chromophore, prepared from pararosaniline (PAR) and sodium sulfite via an addition reaction, reacted with formaldehyde under acidic conditions (pH 1.5) to form red addition products. These products exhibited absorption at 540 nm, which overlapped with the green emission of UCNPs excited at 980 nm. This overlap quenched the green emission through an inner filter effect, while the red emission at 655 nm remained unaffected, enabling sensitive ratiometric detection.

Additionally, UCNPs are attractive nanomaterials that facilitate the NIR-triggered drug release and photodynamic therapy. Jalani et al. synthesized LiYF_4_:Yb^3+^,Tm^3+^ UCNPs via thermal decomposition and coated them with a photocleavable chitosan hydrogel shell to form a nanocomposite hydrogel for controlled drug delivery.^7^ The epoxy groups on the UCNP surface reacted with the amino groups on chitosan, and the chitosan shell was cross-linked by reacting its residual amino groups with a photocleavable cross-linker (PhL) containing succinimidyl and acetylene groups, followed by a click reaction with polyethylene glycol-bisazide to form a stable network. Fluorescein isothiocyanate-labeled bovine serum albumin (FITC-BSA) was used as a model drug to be encapsulated within the chitosan shell before final cross-linking. Upon 980 nm NIR irradiation, the UCNPs upconverted NIR light to UV light, cleaving the PhL and degrading the chitosan shell to release 92% of FITC-BSA within 9 min at 1.8 W/cm^2^, enabling precise on-demand therapeutic delivery with minimal leakage.

### 2.2 Ln-MOFs

Ln-incorporated MOFs (Ln-MOFs) are porous crystalline materials that combine the luminescent and magnetic properties of lanthanides with the tunable porosity and high surface area of MOFs.^44^ Their chemical stability and multifunctionality make them ideal to develop luminescent hydrogels for applications in sensing^9,33,34,45,46^ and biomedical diagnostics.^8,46,47^

Ln-MOFs are highly versatile for developing stimuli-responsive hydrogels due to their tunable luminescence and structural stability. Lian et al. developed an Eu@MIL-116­(Ga)@alginate mixed matrix membrane (MMM) hydrogel, where MIL-116­(Ga) was prepared through a hydrothermal reaction of 1,2,3,4,5,6-benzene hexacarboxylic acid (H_6_mel) and gallium nitrate, followed by postsynthetic incorporation of Eu^3+^ via immersion in a methanol solution.^9^ The nanocomposite hydrogel was formed by integrating Eu@MIL-116­(Ga) nanoparticles with alginate through a Ga^3+^ cross-linking process, where the substrate (paper or fabric) was pre-immersed in a Ga^3+^ solution before dipping into a casting solution containing the MOF–alginate mixture, yielding a uniform hydrogel composite under ambient conditions after rinsing with deionized water. The interface between Eu@MIL-116­(Ga) nanoparticles and alginate was stabilized by coordination between Eu^3+^ and the free carboxyl groups of alginate, enhanced by Ga^3+^-mediated ionic cross-linking, which promoted strong electrostatic interactions and ensured uniform nanoparticle dispersion within the hydrogel network. This MMM achieved a detection limit of 13.4 ppb for mitoxantrone in serum via Förster resonance energy transfer (FRET)-based luminescence quenching (Figure 3a), with a 3 min response and recyclability over four cycles. Additionally, the Eu@MIL-116­(Ga)@Alg MMMs exhibited luminescence quenching with increasing mitoxantrone concentrations in serum under UV irradiation, selective quenching by mitoxantrone among various drugs at 614 nm (Figure 3b), and a selective response in serum with anti-interference capability.

**Figure 3:**
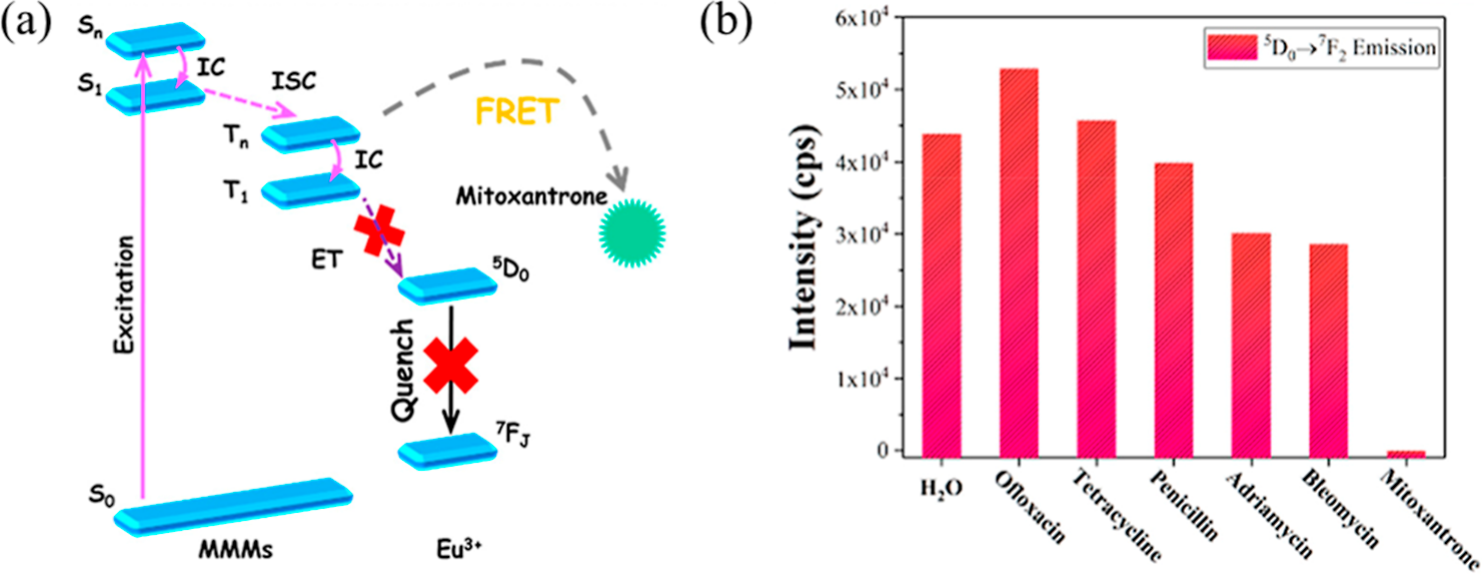
Detection of mitoxantrone using Eu@MIL-116­(Ga)-Alg MMMs via FRET-based luminescence quenching. (a) Schematic of FRET between Eu^3+^ ions and mitoxantrone. (b) Luminescence intensity of Eu@MIL-116­(Ga)-Alg MMMs at 614 nm, showing selective quenching by mitoxantrone among various drugs (reprinted from ref 9 with permission from American Chemical Society, Copyright 2020).

Hydrogels with Ln-MOFs also excel in food safety monitoring through sensitive fluorescence responses. Xu et al. developed a nanocomposite hydrogel by embedding methyl red@lanthanide MOFs (MR@EuMOFs) into a carboxy methyl cellulose (CMC) matrix to detect histamine vapor, a food spoilage indicator.^33^ The nanocomposite hydrogel was prepared by dispersing MR@EuMOFs into a water-phase sodium salt of CMC (CMC-Na), followed by solidification into a hydrogel through ionic cross-linking. Specifically, the CMC-Na solution containing MR@EuMOFs was mixed with agarose, and the mixture was solidified by cooling, forming a physically cross-linked hydrogel where the CMC-Na chains are stabilized by hydrogen bonding and ionic interactions with sodium ions, encapsulating the MR@EuMOF nanoparticles. The interface between MR@EuMOFs and the CMC matrix was primarily stabilized by hydrogen bonding between the hydroxyl and carboxylate groups of CMC and the surface functional groups of MR@EuMOFs, such as the remaining amino groups and the amide bonds formed during MR modification, ensuring a stable nanoparticle dispersion within the hydrogel network. The resulting hydrogel exhibited a color transition from red to blue under UV light upon exposure to histamine vapor, driven by a pH-induced energy transfer mechanism where MR-based fluorescence increased and Eu^3+^-based luminescence decreased, achieving a detection limit of 0.1 μM and a response time of 25 min. Furthermore, the hydrogel enabled the construction of an advanced analytical device using a one-to-two logic gate, where histamine concentration served as the input and the dual fluorescence emissions (MR and Eu^3+^) as outputs, sorting into four logic groups (i.e., NOT (0,1), YES (1,0), PASS 1 (1,1), and PASS 0 (0,0)) for real-time food freshness evaluation.

The adaptability of Ln-MOF-incorporated hydrogels extends to environmental ion detection. Mei and Yan synthesized Eu@MOF-808-TDA and Tb@MOF-808-TDA, using thiodiglycolic acid (TDA) as an intermediate to modify MOF-808 via a postsynthetic modification method.^34^ Tb@MOF-808-TDA was incorporated into a poly­(vinyl alcohol) (PVA) matrix to form a nanocomposite hydrogel, PVA-Tb@MOF-808-TDA, for sensing applications. The hydrogel was prepared by dispersing PVA and Tb@MOF-808-TDA in deionized water, heating the mixture at 95 °C for 2 h to form a homogeneous solution, cooling it to room temperature, and then subjecting it to freeze–thaw cycles (freezing at −18 °C for 12 h, followed by thawing) to induce physical cross-linking. The interface between the nanoparticles and the PVA matrix was primarily stabilized by hydrogen-bonding interactions between the hydroxyl groups of PVA and the surface functional groups of Tb@MOF-808-TDA, such as the uncoordinated carboxyl groups from TDA and the oxygen atoms in the MOFs, ensuring a stable dispersion of the nanoparticles within the hydrogel. The resulting PVA-Tb@MOF-808-TDA hydrogel demonstrated selective detection of aluminum ions (Al^3+^) and uranyl ions (UO_2_^2+^), achieving detection limits of 0.111 ppm for Al^3+^ ions in serum and 0.128 ppm for UO_2_^2+^ in river water, with excellent selectivity and anti-interference ability in real-world samples. Notably, the hydrogel showed good reusability for UO_2_^2+^ detection due to a dynamic quenching and photoinduced electron transfer mechanism, while Al^3+^ detection involved static quenching, competitive adsorption, and ion exchange, limiting its recyclability for Al^3+^ ions. Additionally, Tb@MOF-808-TDA was used to construct numerical recognition systems for multiples of three and four, combining fluorescence signals, hierarchical cluster analysis, and logical gates, enabling applications in advanced analytical devices.

Ln-MOF-incorporated hydrogels further demonstrate potential in advanced biomedical diagnostics. Lian and Yan developed an Eu_2_(BPDC)­(BDC)_2_(H_2_O)_2_ MOF, a mixture of europium­(III) hydroxide (Eu­(OH)_3_), 2,2′bipyridine-3,3′-dicarboxylic acid (H_2_bpydc), 1,4-benzenedicarboxylic acid (H_2_bdc), and H_2_O, integrated with the alginate hydrogel to form a nanocomposite hydrogel for detecting β-lactamase in serum.^8^ The hydrogel was formed by cross-linking the alginate with iron (Fe^3+^) ions, acting as a cation-induced cross-linking agent, creating a stable network through ionic interactions between the negatively charged carboxylate groups of alginate and Fe^3+^ ions. The Eu^3+^-containing MOF nanoparticles were uniformly dispersed within the alginate matrix, with the interface stabilized primarily by coordination interactions between the uncoordinated sites on the MOF and the hydroxyl and carboxylate groups of the alginate, enhancing the mechanical integrity and luminescence properties of the composite. This nanocomposite hydrogel exhibited an “ON–OFF–OFF–ON” luminescence response pattern, where the initial luminescence of Eu^3+^ in the MOF was quenched by Fe^3+^ (OFF state), restored by the binding of penicillamine (a penicillin metabolite) to Fe^3+^ (ON state), and further modulated by β-lactamase activity. The hydrogel achieved a detection limit of 1.25 U mL^–1^ for β-lactamase in serum, demonstrating high sensitivity and selectivity. Research findings include the ability of the hydrogel to detect penicillamine with a limit of detection of 8.74 μM in serum, its excellent anti-interference performance, and its reusability over three cycles after ultrasonic washing, making it a promising tool for the noninvasive diagnosis of penicillin allergy.

Ln-MOF-incorporated hydrogels are also effective for precise environmental pollutant detection. Wang et al. developed a luminescent MOF, Eu^3+^/Tb^3+^@Uio-66-(COOH)_2_/NDC, using 1,2,4,5-benzenetetracarboxylic acid (H_4_btec) and 1,4-naphthalenedicarboxylic acid (1,4-NDC) as mixed ligands, and integrated it into a sodium alginate (SA) hydrogel to form a nanocomposite hydrogel film (LMOF@SA) for the discriminative detection of nitrophenol isomers (*o*-NP, *m*-NP, and *p*-NP).^35^ The nanocomposite hydrogel was prepared by dissolving SA in deionized water to form a solution, which was then mixed with Eu^3+^/Tb^3+^@Uio-66-(COOH)_2_/NDC nanoparticles at varying weight loadings (25, 50, and 75 wt %). This mixture was spread into a film on a glass slide using a scraper and cross-linked by immersing the slide in a 1.5 wt % calcium chloride aqueous solution, where calcium (Ca^2+^) ions facilitated ionic cross-linking with the carboxylate groups of SA, forming a stable three-dimensional hydrogel network. The interface between the MOF nanoparticles and the SA matrix was stabilized through coordination interactions between the uncoordinated carboxyl groups of the MOF and the hydroxyl and carboxylate groups of SA, ensuring uniform dispersion and enhancing the mechanical and luminescent properties of the hydrogel. Research findings include achieving 100% discrimination of *o*-NP, *m*-NP, and *p*-NP isomers at concentrations ranging from 40 to 100 μM in water using linear discriminant analysis (LDA), with the sensor maintaining its performance for binary or ternary NP mixtures at 60 μM and demonstrating semiquantitative analysis from 0 to 80 μM. The LMOF@SA film exhibited excellent portability, stability, and enhanced adsorption capacity, attributed to the porous structure of the hydrogel, making it a practical tool for environmental sensing applications. These studies collectively highlight the high sensitivity, selectivity, and practical applicability of Ln-MOF-incorporated hydrogels in food safety, environmental monitoring, and biomedical diagnostics.

### 2.3 Ln-Nanoclay

Nanoclay, a synthetic layered silicate such as laponite and aminoclay (AC), is widely used in hydrogel formulations due to its excellent dispersibility, biocompatibility, and ability to form physical cross-links with polymers.^10,48^ Ln-incorporated nanoclay (Ln-nanoclay) composites are encapsulated into hydrogels to enhance their structural integrity, luminescent properties, and bioactivity, as well as provide tunable mechanical properties, self-healing, and stimuli-responsive behaviors for sensing^10,39^ and soft actuator applications.^37^

Nanoclay enhances hydrogel properties with its dispersibility and biocompatibility for advanced applications. Li et al. developed a luminescent nanocomposite hydrogel by incorporating acrylamide (AAm) polymerized into polyacrylamide (PAAm) with Ln–ligand (Ln–L) complexes (Ln = Eu^3+^ or Tb^3+^) and AC, a magnesium organosilicate (R_8_Si_8_Mg_6_O_16_(OH)_4_, R = –CH_2_CH_2_CH_2_NH_2_), through in situ copolymerization.^36^ The process involved dispersing AC in water, loading 2,6-pyridinedicarboxylic acid ligands (L) onto AC via electrostatic interactions between carboxyl and amino groups, coordinating Ln^3+^ ions to form Ln–L@AC, and then polymerizing AAm with potassium persulfate and *N*,*N*,*N*′,*N*′-tetramethylethylenediamine (TEMED) at 30 °C for 12 h (Figure 4a). The hydrogel network was cross-linked through the dynamically reversible Ln–L coordination bonds as well as the hydrogen bonds between amino groups on AC and PAAm chains, enhancing the mechanical strength of the hydrogels. The nanocomposite hydrogel achieved over 20-fold stretchability with a fracture stress of 48 kPa, attributed to energy dissipation from de-cross-linking during deformation. Luminescence was improved, with quantum efficiency reaching 32.27% for Eu^3+^ due to reduced water quenching by embedding Ln–L coordination within AC layers, and tunable emission from red to green was observed by adjusting Eu^3+^/Tb^3+^ ratios (Figure 4b). Self-healing was enabled by dynamic coordination and hydrogen bonds, recovering 90% stress after 12 h and allowing stretching to 1700% posthealing (Figure 4c).

**Figure 4:**
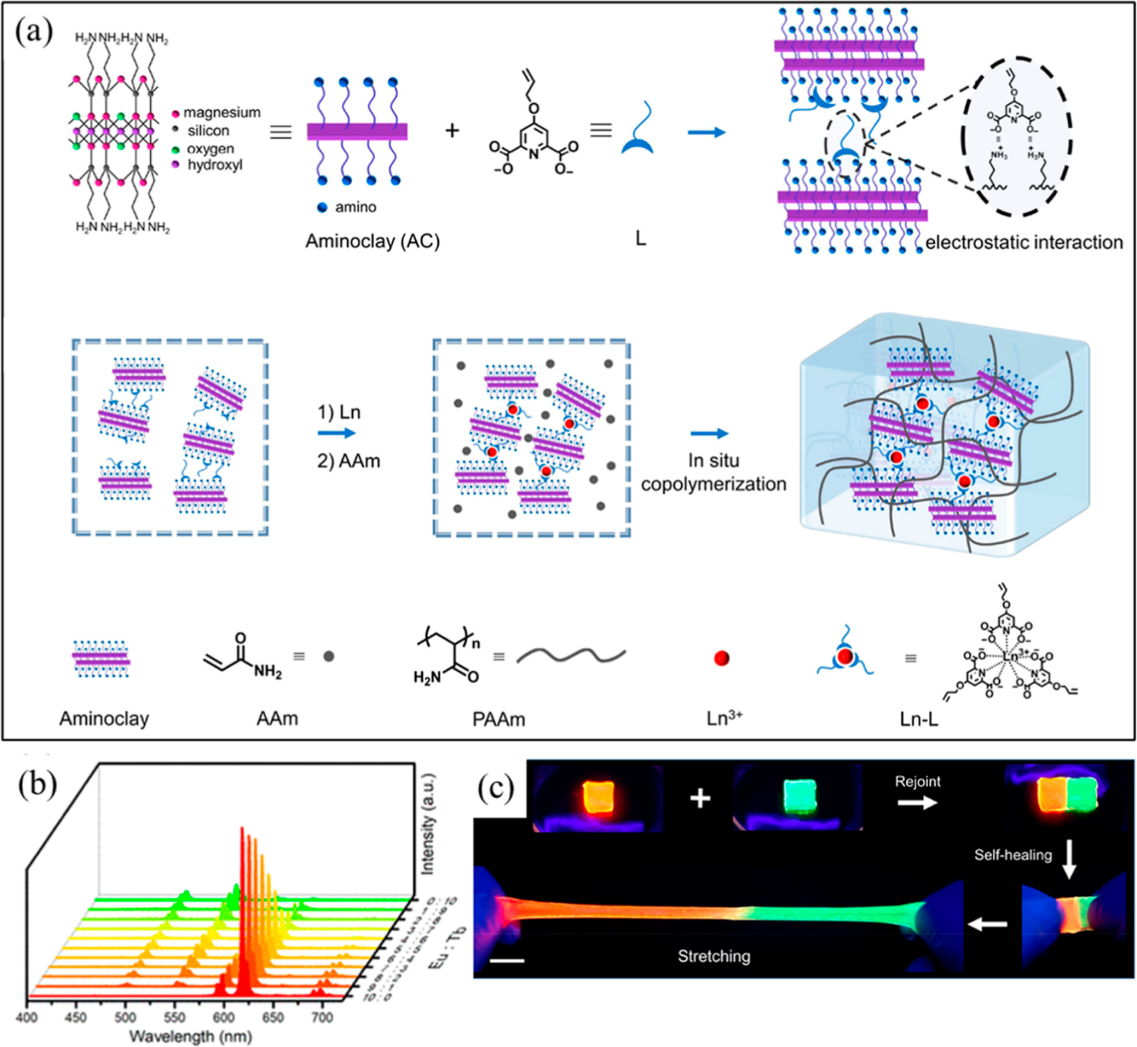
Fabrication and properties of Ln–L@AC nanocomposite hydrogels. (a) Schematic illustration of Ln–L@AC nanocomposite hydrogel formation via in situ copolymerization of AAm with Ln–L@AC. (b) Luminescence spectra of Ln–L@AC hydrogels with varying Eu^3+^/Tb^3+^ ratios, displaying tunable emission from red to green. (c) Photographs of the self-healing process of Eu-L@AC and Tb-L@AC hydrogels, showing 90% stress recovery after 12 h, with stretching to 1700% posthealing (reprinted from ref 36 with permission from American Chemical Society, Copyright 2020).

Laponite (Lap)-based bilayer hydrogels offer advanced thermoresponsive actuation. Wang et al. developed a thermoresponsive bilayer hydrogel actuator with two nanocomposite layers with the copresence of Lap and lanthanide complexes: a poly­(*N*-isopropylacrylamide) (PNIPAM) layer containing Lap and Tb^3+^ complexes (Lap-Tb-L_3_) and a poly­(*N*,*N*-dimethylacrylamide) (PDMA) layer containing Lap and Eu^3+^ complexes (Lap-Eu-L_3_), with 2,6-pyridine dicarboxylic acid (L_3_) as the ligand in the complexes (Figure 5a).^37^ The hydrogel was prepared through sequential photopolymerization, where Lap was exfoliated in water, mixed with Tb^3+^ or Eu^3+^ and L_3_ to form Lap-Tb-L_3_ or Lap-Eu-L_3_, and then combined with NIPAM or DMA monomers, *N*,*N*′-methylenebis­(acrylamide) (MBAA) cross-linker, and 2,2-diethoxyacetophenone (DEAP) photoinitiator, followed by UV curing of the PNIPAM-Lap-Tb-L_3_ layer first and then the PDMA-Lap-Eu-L_3_ layer cast and cured atop it. Lap acted as physical cross-linkers, forming hydrogen bonds and electrostatic interactions with PNIPAM and PDMA chains, while MBAA provided covalent cross-linking, enhancing tensile strength as shown in stress–strain curves. The mechanical analysis clearly demonstrated that the incorporation of both Lap and Ln·L_3_ introduced a highly compact and interconnected internal network within the Ln-L@AC hydrogel. While pure Ln–L or AC hydrogels generally formed relatively loose and less efficient cross-linking frameworks, the dual presence of Lap nanosheets and Ln·L_3_ complexes acted as multifunctional linkers that significantly reinforced the polymer matrix. This dual-cross-linking effect markedly increased the cross-linking density, leading to superior load transfer and energy dissipation during deformation. As a result, the Ln-L@AC hydrogel achieved a much higher tensile stress and maintained large fracture strains compared to single-component hydrogels, confirming that its compact internal structure underpins its outstanding mechanical strength and resilience.

**Figure 5:**
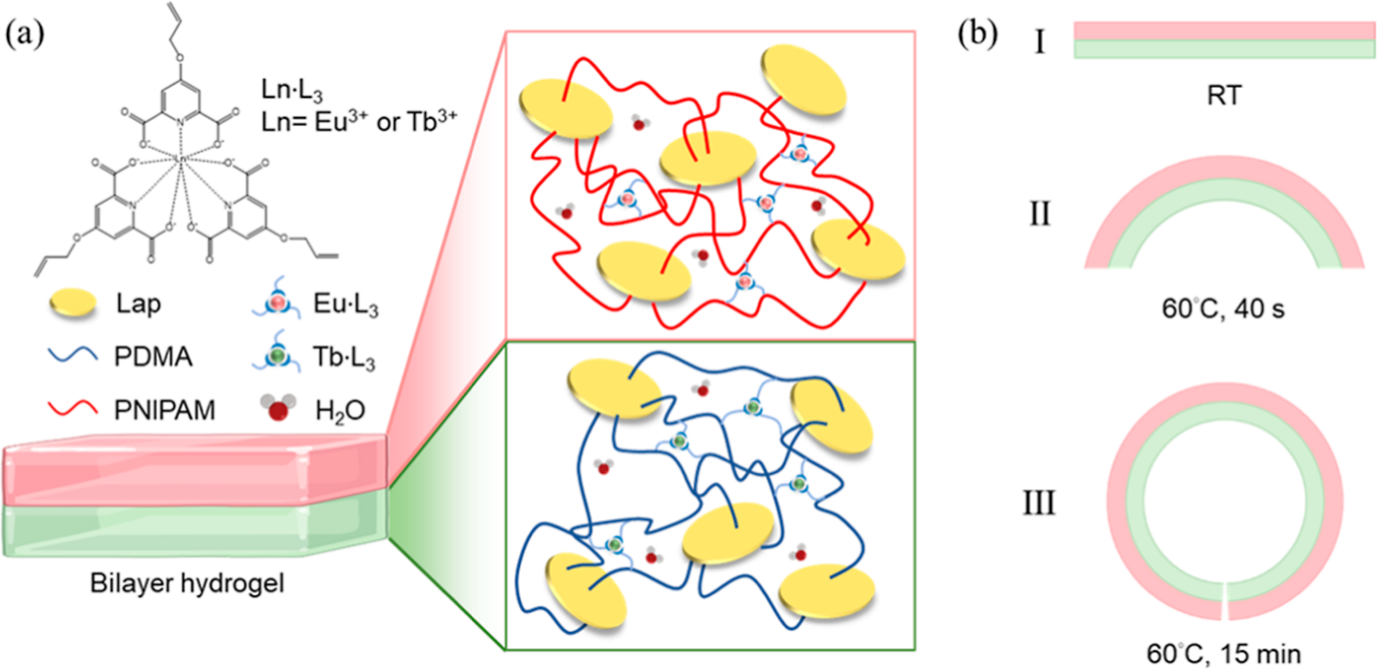
Schematic illustration of (a) polymerized PNIPAM-Lap-Tb-L_3_ and PDMA-Lap-Eu-L_3_ bilayer hydrogel as a thermal-responsive smart luminescent actuator. (b) Bilayer hydrogel was placed at room temperature (I) and heated in 60 °C water for 40 s (II) and 15 min (III).

Above the lower critical solution temperature (LCST, 32 °C) of PNIPAM, the PNIPAM layer dehydrated and contracted, driving reversible bending to a C shape (175°) within 40 s and bending up to 371° within 15 min (Figure 5b), while the PDMA layer remained swollen, with full unbending achieved in 4 h at 0 °C. Luminescence intensified upon heating, with the PNIPAM-Lap-Tb-L_3_ layer emitting green light at 544 nm, increasing by 29.6% with a lifetime of 1.47 ms, and the PDMA-Lap-Eu-L3 layer emitting red light at 615 nm, increasing by 20.4% with a lifetime of 1.35 ms, due to reduced water quenching. The robust mechanical properties and thermal responsiveness of the hydrogel enabled complex three-dimensional (3D) deformations, such as flower-shaped actuators for reversible capture and release of objects, demonstrating potential in smart manipulators for bionic applications.

Lap also supports luminescent hydrogels for precise sensing. Chiang et al. developed luminescent nanocomposite double-network hydrogels by incorporating terbium-containing laponite (Tb^3+^@Lap) into polyethylenimine-modified gelatin (PG) and polydextran aldehyde (PDA) networks, formed via dynamic imine bonds between the amine groups on PG and aldehyde groups on PDA, supplemented by hydrogen bonds, electrostatic interactions, and Tb^3+^–carboxylate coordination bonds. The PG/PDA/Tb^3+^@Lap hydrogel exhibited a higher storage modulus, a lower swelling ratio, and slower degradation compared with the PG/PDA hydrogel. The dynamic bonds in the PG/PDA/Tb^3+^@Lap hydrogel network enabled shear-thinning, self-healing, and injectability, facilitating their processing through electrospinning and 3D printing to fabricate customizable structures on a macroscale. Tb^3+^@Lap imparted green luminescence at 544 nm for selective copper ion sensing with a detection limit of 1.12 μM and high sensitivity, driven by luminescence quenching via disrupted Tb^3+^ coordination.^10^ The same research group further advanced the luminescent nanocomposite hydrogels by incorporating organic ligands to enhance the luminescence and detection capabilities of the hydrogels.

Zeng et al. incorporated europium-containing laponite (Eu^3+^@Lap) or 2-thiophenyltrifluoroacetone (TTA)-modified Eu^3+^@Lap (Eu^3+^(TTA)@Lap) into the dynamic PG/PDA network (Figure 6a).^25^ The PG/PDA/Eu^3+^(TTA)@Lap hydrogel exhibited stronger red luminescence at 615 nm with a photoluminescence quantum yield of 12.9% compared to 7.1% for the PG/PDA/Eu^3+^@Lap hydrogel due to the antenna effect of TTA (Figure 6b). PG/PDA/Eu^3+^(TTA)@Lap-lyophilized hydrogel distinguished formaldehyde from volatile organic compounds via LDA (Figure 6c), achieving a calculated detection limit of 39 ppb. The detection of formaldehyde in the PG/PDA/Eu^3+^(TTA)@Lap-lyophilized hydrogel was attributed to the competitive coordination of formaldehyde with Eu^3+^, displacing TTA and quenching luminescence, with reversible luminescence restoration upon TTA recoordination. Overall, incorporating TTA onto Eu^3+^@Lap to form Eu^3+^(TTA)@Lap introduced additional π–π stacking interactions between thiophene rings, resulting in a more compact microstructure and enhanced mechanical strength of the PG/PDA/Eu^3+^(TTA)@Lap hydrogel compared with the PG/PDA/Eu^3+^@Lap hydrogel. Moreover, the TTA ligand efficiently harvested the excitation energy and transferred it to embedded Eu^3+^ ions via the antenna effect to improve the luminescence efficiency. Consequently, PG/PDA/Eu^3+^(TTA)@Lap hydrogels exhibited superior sensing performance, particularly in selectively distinguishing formaldehyde from other VOCs.

**Figure 6:**
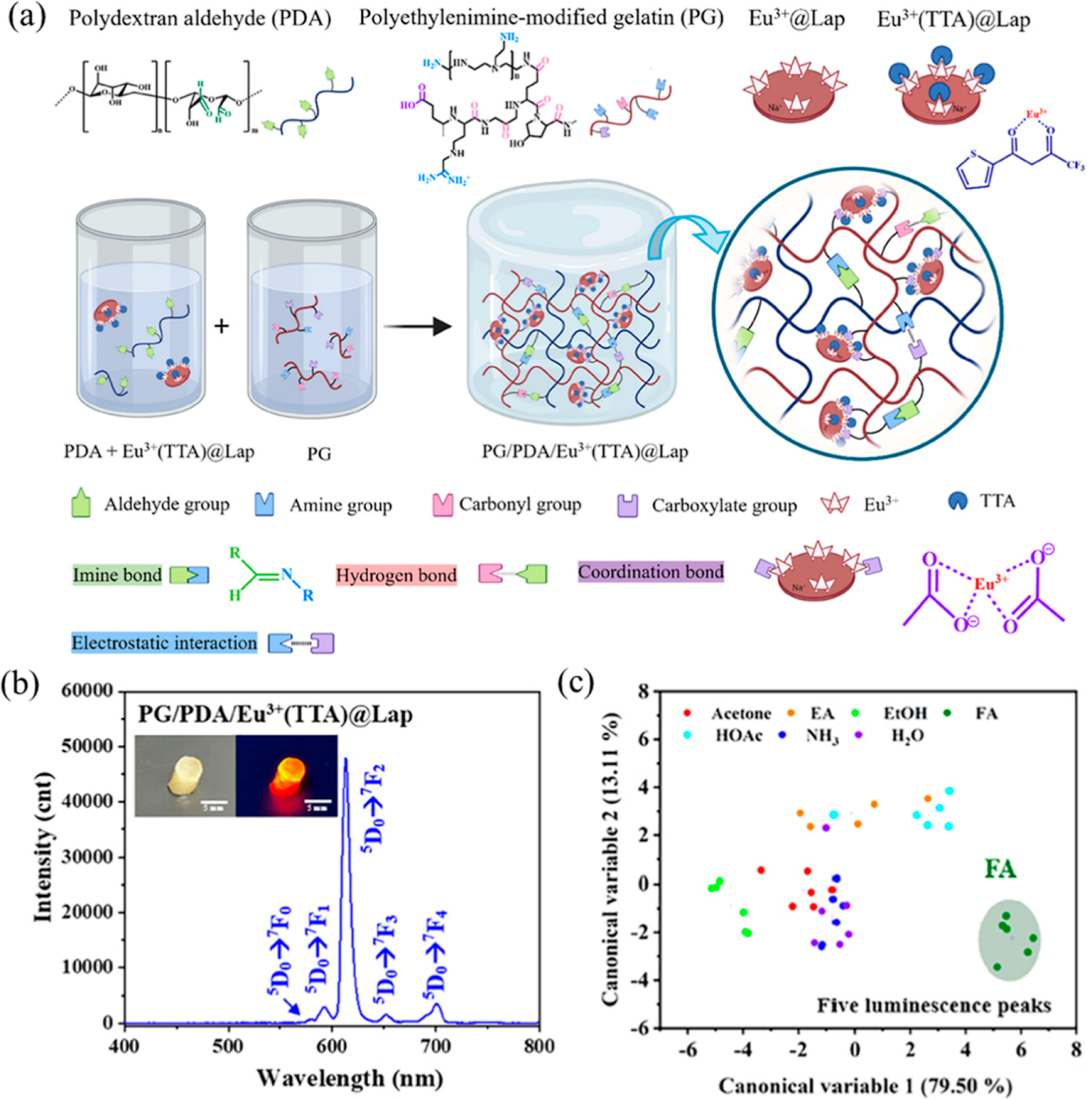
Structure, luminescence, and VOC sensing of PG/PDA/Eu^3+^(TTA)@Lap hydrogels. (a) Schematic illustration of the components and interactions in the PG/PDA/Eu^3+^(TTA)@Lap hydrogel network. (b) Luminescence spectrum of PG/PDA/Eu^3+^(TTA)@Lap-lyophilized hydrogel under 360 nm excitation, displaying enhanced red emission at 615 nm. (c) Canonical score plot for gas sensing of PG/PDA/Eu^3+^(TTA)@Lap-lyophilized hydrogel obtained from LDA by analyzing through five luminescence peaks (579, 591, 611, 650, and 698 nm) (reprinted from ref 25 with permission from Wiley-VCH, Copyright 2024).

Lap enables thermoresponsive and luminescent hydrogels for smart applications. Dong et al. developed co-cross-linked luminescent hydrogels through in situ polymerization of *NIPA* with Lap and lanthanide complexes (Ln–L_3_) containing Eu^3+^ or Tb^3+^ coordinated with 2,6-pyridinedicarboxylic acid.^38^ The hydrogel was synthesized by dissolving Eu–L_3_ or Tb–L_3_ in water, adding Lap and NIPA monomers, stirring for 3 h, and then incorporating *N*,*N*,*N*′,*N*′-tetramethylethylenediamine (TEMED) and potassium peroxodisulfate (KPS) as initiators, followed by gelation at room temperature for 24 h in an oxygen-free environment. Lap and Ln–L_3_ complexes served as cross-linkers, with Lap forming physical cross-links through hydrogen bonds and electrostatic interactions with PNIPAM chains, while Ln–L_3_, containing polymerizable allyl moieties, provided chemical cross-linking to strengthen the hydrogel structure. The hydrogels exhibited elongation at break up to 800%, transmittance above 80% at 25 °C, and tunable emission colors by varying Eu^3+^/Tb^3+^ molar ratios, showing red emission at 615 nm for Eu^3+^ and green emission at 544 nm for Tb^3+^. Above the LCST of PNIPAM, the hydrogel dehydrated, becoming opaque, with NC-3%-Eu-L_3_ and NC-5%-Eu-L_3_ hydrogels showing five times luminescence intensity increase at 50 °C due to reduced water quenching, while NC-8%-Eu-L_3_ showed a 2-fold increase due to hindered hydrophobic transitions from a higher Lap content. These thermoresponsive and conductive properties enabled applications in intelligent display devices and smart sensors.

### 2.4 Ln-HAp

HAp, a naturally occurring mineral in bone, can be doped with lanthanide ions to create luminescent biomaterials.^49^ Ln-incorporated HAp (Ln-HAp) can be introduced into hydrogels to enhance their bioactivity and enable multifunctional applications in bone regeneration^23,50^ and imaging.^50,51^

Recent studies have demonstrated the versatility of the Ln-HAp-containing nanocomposite hydrogels by leveraging dynamic cross-linking chemistries and advanced fabrication techniques to achieve tailored properties for diverse applications. For instance, Su et al. developed a series of luminescent nanocomposite hydrogels by cross-linking amine-functionalized Ln-HAp (Ln-HAp@L) with PDA through dynamic imine bonds.^11^ The preparation involved synthesizing surface-functionalized Ln-HAp@L by doping HAp with Ln ions (i.e., Eu^3+^, Tb^3+^, or dysprosium (Dy^3+^)) via a precipitation method, followed by capping with amine-terminal ligands, such as nitrilotriacetic acid, di­(2-picolyl)­amine (DPA), and bisphosphonate. These Ln-HAp@L nanoparticles were then mixed with PDA, forming a hydrogel network where the amines on Ln-HAp@L reacted with the aldehydes of PDA to form dynamic imine bonds (Figure 7a). Rheological analysis confirmed that Eu^3+^ incorporation was essential, as PDA/Eu-HAp exhibited a solid-like behavior in contrast to the liquid-like state of PDA or PDA/HAp. Notably, PDA/Eu-HAp@NTA displayed a storage modulus (∼1085 Pa) dramatically higher than that of dextran/Eu-HAp@NTA (∼37 Pa), highlighting that strong Eu^3+^ coordination and imine bond formation with surface ligands were critical for constructing a compact, mechanically robust hydrogel network. This cross-linking chemistry, driven by both imine bonds and metal–ligand interactions, enabled tunable luminescence, microstructures, and mechanical properties of nanocomposite hydrogels.

**Figure 7:**
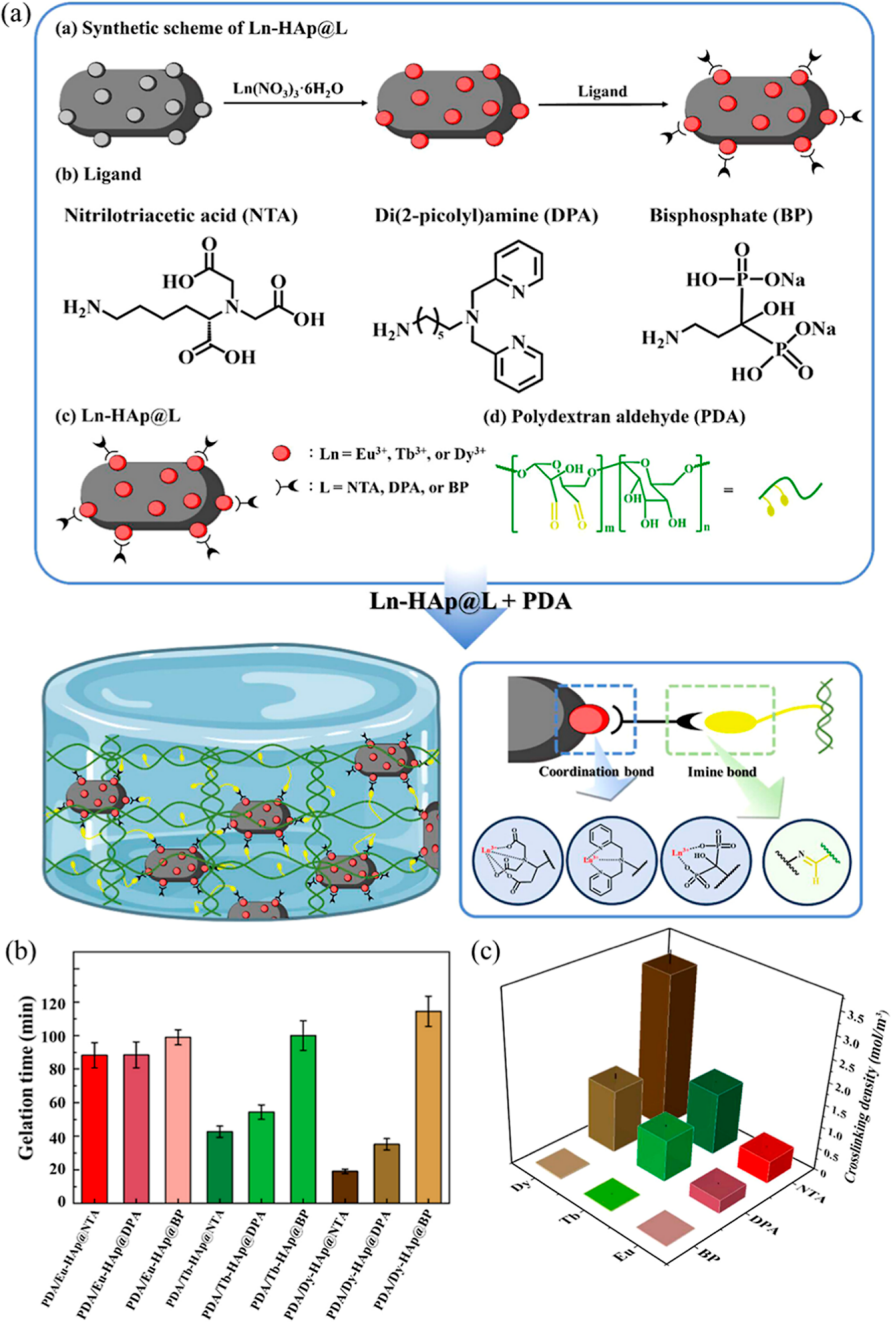
Preparation and properties of PDA/Ln-HAp@L nanocomposite hydrogels. (a) Schematic illustration of the synthesis of Ln-HAp@L and its cross-linking with PDA via dynamic imine bonds. (b) Gelation times and (c) cross-linking density of PDA/Ln-HAp@L hydrogels. (reprinted from ref 11 with permission from American Chemical Society, Copyright 2024).

The study revealed that PDA/Dy-HAp@NTA hydrogels exhibited the fastest gelation due to stable Dy–NTA coordination (Figure 7b) as well as the highest cross-linking density of the hydrogel network (Figure 7c). Additionally, these hydrogels demonstrated enhanced self-healing efficiency, especially for NTA-based hydrogels with Dy^3+^ ions, attributed to the dynamic imine and coordination bonds, alongside shear-thinning and injectable properties. These characteristics make the hydrogels suitable for differentiating VOCs (e.g., acetic acid, ammonia, and formaldehyde) via luminescent fingerprints analyzed by LDA, as well as for potential biomedical applications such as 3D printing and bioimaging.

Complementing these advances, Leu Alexa et al. explored the potential of cerium (Ce)-doped HAp in GelMA hydrogels to enhance bone regeneration through 3D printing technology.^23^ The resulting hydrogel was photo-cross-linked under UV light (365 nm) to form a covalent network, with HAp physically embedded within the GelMA matrix to enhance mechanical properties and bioactivity. The study found that scaffolds with 3% HC5 (0.5% Ce doping) and 30% GelMA exhibited the highest biocompatibility, cell viability, and proliferation of MC3T3-E1 murine pre-osteoblasts. Additionally, these scaffolds supported osteogenic differentiation, with increased osteopontin and osterix expression after 28 and 14 days, respectively, and demonstrated optimal 3D printability with high structural integrity, making them promising for bone tissue engineering applications.

### 2.5 Ln-Carbon-Based Materials

Carbon-based materials (e.g., carbon quantum dots (CQDs)^12^ and graphene oxide^52^) can be doped with lanthanide ions to offer unique electrical, luminescent, and photothermal properties. When incorporated into hydrogels, these materials enable applications in anticounterfeiting^12,53^ and environmental sensing.^54,55^

By the integration of Ln-functionalized CQDs into a polymer matrix, advanced hydrogels can be designed for precise ion sensing. Wen et al. developed a stepwise assembly protocol for Eu^3+^- or Tb^3+^-functionalized CQD nanocomposite hydrogels using alginate as the polymer matrix for Ca^2+^ ion sensing (Figure 8).^12^ The CQDs were functionalized with ethylenediaminetetraacetic acid (EDTA) by dispersing CQDs in bicarbonate buffer (pH 9.6), adding europium­(III) nitrate (Eu­(NO_3_)_3_) or terbium­(III) nitrate (Tb­(NO_3_)_3_), and reacting with EDTA in dimethyl sulfoxide for 48 h, forming CQD–EDTA–Ln^3+^ complexes via coordination bonds. The nanocomposite hydrogel was prepared by mixing CQD–EDTA–Ln^3+^ complexes with alginate, dropping onto a glass slide, and drying at room temperature for 24 h. The dried film was immersed in a CaCl_2_ solution for 45 s to induce ionic cross-linking, where Ca^2+^ coordinated with the guluronic acid units of alginate, forming a three-dimensional network encapsulating CQD. The CQD–polymer interface in the network can be stabilized with hydrogen bonding and van der Waals forces. The resulting hydrogels exhibited characteristic red (Eu^3+^, 617 nm) and green (Tb^3+^, 545 nm) emissions under 363 nm UV excitation, with luminescence lifetimes of 891 μs (Eu^3+^) and 975 μs (Tb^3+^). The hydrogels detected Ca^2+^ with limits of 0.84 μM (Eu^3+^) and 0.92 μM (Tb^3+^) via static quenching, where the emission intensity decreased as Ca^2+^ concentration increased from 10^–3^ to 10^–7^ M. The system was applied in anticounterfeiting films, demonstrating potential for chemical sensing and environmental monitoring.

**Figure 8:**
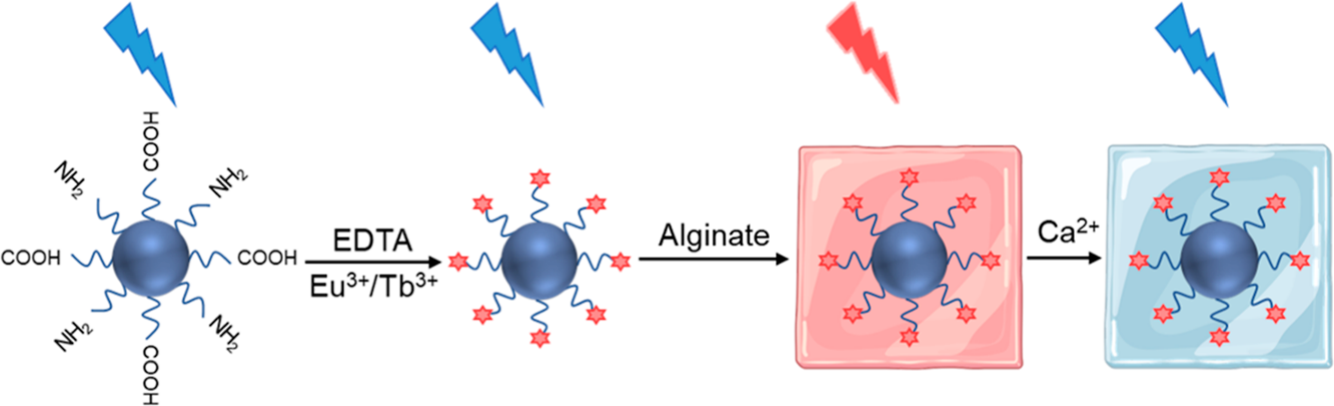
Schematic illustration of the assembly of CQD–lanthanide–alginate hydrogel and its luminescence switching mechanism for Ca^2+^ detection, where Eu^3+^- or Tb^3+^-functionalized CQDs were used.

Beyond sensing, Ln-imprinted carbon-based composites can achieve selective ion separation through tailored binding sites. Li et al. developed a Gd^3+^-imprinted chitosan/carbon nanotube composite (IIP-CS/CNT) for selective adsorption of Gd^3+^ from mixed rare earth ion solutions.^26^ The IIP-CS/CNT was synthesized via a “surface deposition–cross-linking” method, where chitosan was dissolved in acidic solution, deposited onto CNTs, and cross-linked with glutaraldehyde in the presence of Gd^3+^ templates to form a well-defined coating structure. After template removal, cavities complementary to Gd^3+^ in size were created within the chitosan layer. The composite was magnetized by blending with silica-coated magnetite nanoparticles (SiO_2_@Fe_3_O_4_), which adhered to the IIP-CS/CNT network under an external magnetic field, enabling easy retrieval without centrifugation or filtration. The IIP-CS/CNT showed a saturation adsorption capacity of 88 mg g^–1^ for Gd^3+^ at 303.15 K, significantly higher than those of reported rare earth ion-imprinted adsorbents. Selectivity coefficients related to lanthanum­(III) (La^3+^) and Ce^3+^ ions were 3.50 and 2.23, respectively, attributed to the imprinted sites tailored to the ionic radius of Gd^3+^ (0.938 Å). The composite maintained a stable adsorption capacity over five consecutive cycles after regeneration with hydrochloric acid, demonstrating excellent reusability. This work highlights a facile fabrication of magnetic imprinted nanocomposites for efficient Gd^3+^ separation.

## 3 Conclusions and Outlook

This article delivers a comprehensive overview of Ln-containing nanocomposite hydrogels, uniquely categorizing them by the nanomaterial type to illuminate how structure governs properties and function. Through the strategic selection of lanthanide species, hydrogel matrices, and synthesis strategies, these systems emerge as versatile platforms for applications spanning sensing, biomedicine, and information security. Nevertheless, challenges (e.g., luminescence quenching, nanoparticle aggregation, limited long-term stability, biocompatibility risks, and scalability barriers) must be overcome to realize their full potential.

Future research is expected to focus on advanced synthesis strategies to stabilize luminescence by embedding lanthanide ions within hydrophobic microenvironments and leveraging energy transfer mechanisms, achieve precise control of dispersion and morphology of Ln-containing nanomaterials within hydrogel matrices for improving mechanical performance and ensuring long-term structural integrity, and introduce innovations in cross-linking and self-healing chemistry to enhance the structural performance. Additionally, the integration of AI-guided design, coupled with efforts to improve biocompatibility through effective encapsulation or the design of inherently safer lanthanide compounds, will be essential to accelerate the discovery and optimization of next-generation Ln-containing nanocomposite hydrogels for biomedical translation. Finally, addressing scalability, cost, and regulatory issues will be pivotal for practical deployment.

Taken together, these directions point toward a promising pathway for advancing Ln-containing nanocomposite hydrogels into impactful, real-world applications. Ongoing interdisciplinary research is set to drive the advancement of these materials, with emerging innovations steadily uncovering their potential for widespread global applications.
